# Study on Benefit Coordination of Supply Chain Network Based on Green Development

**DOI:** 10.3390/ijerph16081458

**Published:** 2019-04-24

**Authors:** Xiong Gao, Yuhong Wang

**Affiliations:** School of Business, Jiangnan University, Wuxi 214122, China; wo.a17@foxmail.com

**Keywords:** sustainability, green index, leading manufacturer, supply chain network, network profit

## Abstract

Based on Stackelberg’s master–slave game theory and green index decision-making conditions, this paper studies the benefit coordination of a supply chain network composed of a business flow network and logistics network, discusses the decision-making behavior of the main body of the supply chain network under the performance of green contracts or speculative behavior, respectively, and further constructs the supply chain network collaborative benefit coordination model under the guidance of a manufacturer considering a green development index. The supply chain network interest coordination model analyzes the relationship between the dominant manufacturer behavior and the supply chain network green index and network profit. The results show that fulfilling green contracts helps improve the profitability and sustainability of supply chain networks. A counter-intuitive but interesting result is that the dominant manufacturers increase the cost-sharing ratio or penalties of the logistics network, which will reduce the profit level and green index of the logistics network, and increase the cost-sharing ratio or punishment of the suppliers. Strength will increase the profitability and green index of the logistics network. Finally, we validate the relevant conclusions of the model through numerical simulation analysis.

## 1. Introduction

Considering the huge demand for green development of the supply chain, many scholars both here and abroad have done a lot of research on the benefit distribution of the green supply chain. For example, Qu et al. (2018) Considered the influence of heterogeneity of consumers on product demand uncertainty, and constructed a two-level risk decision model of the green supply chain based on hybrid conditional value at risk criteria. Finally, the effectiveness of the model was verified by numerical simulation analysis [1]. Song et al. (2018) established two kinds of green supply chain game models considering consumers sensitivity to green products. By comparison with the centralized control and decentralized decision-making game model, it was concluded that the revenue-sharing contract model constructed in this paper has more advantages than the centralized control and decentralized decision-making game model [2]. Hong et al. (2018) constructed a cooperative contract model of green product supply chain considering the environmental responsibility in a two-echelon supply chain, and studied the impact of a cooperative contract on the environmental performance [3]. Xu et al. (2018) introduced a low-carbon development and environmental governance into a supply chain coordination model for decentralized and centralized markets based on the improved Shapley value method, thus establishing a theoretical model for the income distribution mechanism of the centralized market [4]. Xiao et al. (2018) considered the relationship between enterprise efficiency and environment, studied the feasibility of green supply chain management implemented by the government as an external driving force, established a mixed guidance model of green supply chain management, and further discussed the mechanism of government encouraging enterprises to implement green supply chain management [5]. Zhu et al. (2017) considered the bargaining behavior of customers and established a two-channel closed-loop supply chain model composed of manufacturer, retailer and network recycling platform by using Stackelberg game theory [6]. Bai et al. (2017) constructed profit models of cooperative and non-cooperative green supply chains considering the cooperation between core manufacturers and retailers of environmental input. The effects of different modes on decision variables such as environmental input and product price were discussed [7]. Dai et al. (2017) used game theory to analyze two typical cooperative behaviors of supply chain members: cartelization and cost-sharing contract, and compared cartelization, cost-sharing contracts and the non-cooperation benchmark [8]. Du et al. (2017) considered the fair concerns of suppliers and manufacturers on the sustainable development of green technology innovation, and analyzed the supply chain model of a supplier and a manufacturer [9]. Chen et al. (2016) considered the government’s incentive policy, used contract theory and Nash negotiation theory to construct three decision-making models of concentration, decentralization and negotiation coordination of a wood-based panel green supply chain, and established a revenue-sharing–cost-sharing contract negotiation coordination mechanism. Finally, relevant conclusions were drawn by numerical analysis [10]. Jiang et al. (2015) considered four kinds of green supply chain game models of product greenness. Based on the four game models, the differences among product greenness, product price and wholesale price were compared and analyzed, and a revenue-sharing contract game model was established [11]. Debabrata Ghosha and Shah et al. (2015) constructed a green supply chain cost-sharing contract model through game theory, revealing how product greening levels, prices and profits are affected by cost-sharing contracts within the supply chain [12]. Qi et al. (2014) established a green-supply chain knowledge-sharing principal-agent model based on principal-agent theory, discussed the impact of various parameter changes on the conditions for establishing knowledge-sharing contracts, proposed corresponding strategies, and established a knowledge-sharing mechanism [13]. Hsueh (2014) proposed a revenue sharing contract embedded in corporate social responsibility for the coordination problem of a two-tier supply chain that improves the social responsibility performance of supply chain enterprises by using improved revenue sharing contract [14]. Giovanni et al. (2014) established a performance concept model based on a green supply chain on the basis of existing literature on the impact of environmental management, and suggested the emissions trading system is not better than the emissions trading on the environment and has a better economic performance [15]. Faccio et al. (2014) considered the problem of the innovative sustainable closed-loop supply chain (CLSC), and introduced a linear programming model to minimize the total cost of the supply chain. Through a parameter study, the economic sustainability of the CLSC model and the forward supply chain model (FWSC) was analyzed from two perspectives [16]. Yang et al. (2014) studied the multi-criteria optimization conditions and network equilibrium conditions of decision makers in a multi-level closed-loop supply chain network. A multi-criteria decision equilibrium model of a closed-loop supply chain network considering environmental indicators was established. Finally, the equilibrium results of the decision conditions in the equilibrium model under different environmental emission weights were compared and analyzed by an example [17]. Barari et al. (2012) set up a coordinated green development model between manufacturer and retailer, including greenness and economic benefits, constructed a cooperative alliance between environment and supply chain benefits, and finally used evolutionary dynamics to find the best economic benefits and the most stable point [18]. Debabrata Ghosh and Janat Shah (2012) established a game theory model that considers product greens, revealing the impact of the supply chain channel structure on product green level, selling price and supply chain profit [19]. Perotti et al. (2012) discussed the specific practices of implementing a green supply chain and the impact of taking advice on each approach on the performance of third-party logistics enterprises, which helps to better understand the relationship between the green supply chain and corporate performance [20]. Yang et al. (2011) constructed the revenue sharing contract to construct a supply chain network equilibrium model with stochastic demand, discussed the impact of demand disruption caused by emergencies on the network equilibrium state, and used numerical examples to verify the validity of the model [21]. Ni et al. (2010) analyzed the different interactive game models between enterprises and suppliers, obtained the equilibrium state of the game, and determined the optimal allocation of social responsibility based on the performance criteria of economic and corporate social responsibility [22]. Wang et al. (2010) quantified the impact of environmental performance on profit distribution by using the multi-level fuzzy comprehensive evaluation method, and proposed a profit distribution scheme of green supply chain based on the optimal shape value [23]. Zhu et al. (2010) considered three systems of enterprise operation, and applied the theory of system dynamics to establish the dynamic model of enterprise implementation of green supply chain management [24].

In summary, numerous scholars have invested in the research of sustainable development of a green supply chain, but the distribution of network benefits, as a research subject that discusses environmental factors, is rarely involved. At the same time, few studies have included environmental variables as supply chain network stakeholders’ decision-making conditions into the supply chain benefit distribution model. The contribution of this paper has three aspects: First, we discuss the benefits of supply chain network coordinated and sustainable development through green index (characterization of environmental impact of supply chain product and process) to build a supply chain network coordination function as a decision variable conditions. Second, we construct the overall interest coordination and network sustainable development of the supply chain network under the conditions of fulfilling the green contract or violating the green contract from the perspective of the integration of the business network, the logistics network and the information flow network. Finally, our analysis shows that the supplier-led supply chain network entity can obtain greater benefits under the conditions of fulfilling the green contract and is beneficial to maintain the sustainable development of the supply chain. Through the research of the article, we hope to provide theoretical and operational guidance for the green development of the supply chain.

## 2. Problem Method Description and Basic Assumptions

According to the actual situation of supply chain development, the supply chain network is divided into three sub-network entities: business flow network S, logistics network L and information flow network F. The business flow network is mainly composed of suppliers and manufacturers. Supply chain nodes form a community of interests through the production of environmentally friendly products. In order to maximize the benefits of supply chain nodes and the stability of the supply chain network, each node highlights its advantages and strengthens information, funds, etc., under the coordination of manufacturers. Complementary resource sharing reduces the impact of supply chain activities on the environment, and achieves supply chain equilibrium and green development. Green index serves as a link between supply chain nodes and plays a decisive role in supply chain balance and green development. Therefore, this article focuses on the distribution mechanism of supply chain network benefits under the conditions of green index among supply chain nodes.

### 2.1. Description of the Problem

**Figure 1:**
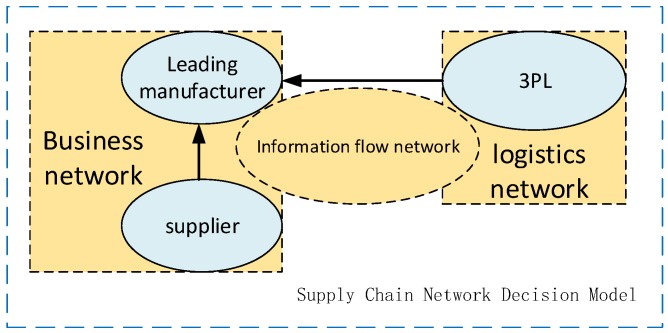
Supply chain network decision model considering a green index.

“Sustainability” will surely become one of the key topics in all fields of society in the 21st century. The focus on environmental issues has risen from the micro level of enterprises to the middle level of supply chain networks, forcing supply chain networks to not only pursue profitability, but also pursue environmental sustainability (This paper studies the use of a green index to characterize environmental sustainability, primarily in the sustainability of products and processes). How to realize supply chain decision-making that is beneficial to the external environment and the sustainable development of the supply chain to maximize the benefits of the supply chain entities and the entire supply chain network is the main problem that needs to be urgently solved in the supply chain. This paper builds a supply chain decision network from a supplier, a dominant core manufacturer, and a third-party logistics company, as shown in Figure 1.

In order to maximize the benefits of supply chain based on a green index, in the supply chain network decision model, manufacturers as the leader of the supply chain network, in each stage of the game, promise to provide suppliers and third-party logistics companies a cost-sharing ratio to maintain the green index (θ,δ)  and satisfy 0≤θ,δ≤1. Suppliers and third-party logistics companies choose whether to implement a supply chain green contract based on the revenue of their normal transactions. After accepting the green contract, they need to make a green index commitment agreed by the manufacturer and bear the cost of implementing the green contract. After the end of the trading period, the manufacturer “perceives” that the supplier and the third-party logistics company have not reached the green index agreed by the manufacturer. The manufacturer will not only bear the green index maintenance cost of the supplier and the third-party logistics company, but also punish it.

### 2.2. Method Description

This paper combs and explores the research progress of the green development of a supply chain, and coordination of interests of the supply chain network at home and abroad, by using a literature research method to find out the weaknesses of the research and grasp the latest research trends. Then, based on Stackelberg’s master–slave game theory, the paper analyses the game behavior of a business flow network, logistics network and decision-makers under the conditions of fulfilling green contracts and speculation. Then, the benefit coordination model of supply chain network considering the green index is constructed. Then, through comparative analysis, the benefit distribution of decision-makers in supply chain networks under the performance of a green contract and speculative behavior is compared. It is concluded that the performance of a green contract can obtain greater benefits and is beneficial to maintain the green development of a supply chain network. Finally, the models are validated by the simulation test method.

### 2.3. Model Basic Assumptions

**Hypothesis** **1.** *Let $U=\left\{u_{1i},u_{2i},u_{3i}\right\}$ be a collection of various stakeholders in the supply chain network, including suppliers, manufacturers and third-party logistics, and (i=1,2,⋯,n) is the interest subject. The article assumes that each stakeholder group only considers the existence of one stakeholder. The realization of the sustainable value chain of the supply chain network environment is accomplished through the construction of a green index (sustainability of the main products and processes in a supply chain) among the subjects. The change of the green index will not only affect the maximum interests of each subject, but also affect the balance of the supply chain network interest chain. Therefore, each subject of the supply chain network takes the maximization of benefits as the premise of decision-making.*

**Hypothesis** **2.** *Considering that the supply chain network is a complex network system formed by superposition and coupling of a business flow network, a logistics network and an information flow network, wherein the business flow network is composed of a manufacturer and a supplier. The manufacturer is the maker and supervisor of the supply chain network green index contract, with the right to control and coordinate the entire supply chain network. In this process, we assume that the risk is neutral. On this basis, after the supplier and the manufacturer have concluded a green contract, they promise that each period of the normal transaction will purchase green orders from the supplier at price ω, while the supplier will produce the order at cost $C_{1}$, and then the manufacturer sells to the end customer at retail price $P_{S}$. After the business flow network and the logistics network conclude the green contract, each phase of the normal transaction is promised to realize the material transfer activities of the business flow network with the unit logistics price $P_{l}$ and the unit logistics cost $C_{2}$.*

**Hypothesis** **3.** *As the green index is the perfect public equilibrium of an indefinite game, in the process of the game between the two parties, the supplier can observe the output $D_{s}$ of its own compliance, green index $e_{s}$ and green cost $C^{S}\left(e_{s}\right)$, and the manufacturer can only observe the output $D_{S}$. The logistics network can observe its own compliance output $D_{l}$, green index $e_{l}$ and green cost $C^{l}\left(e_{l}\right)$, and the manufacturer can only observe the output $D_{l}$. Therefore, there is a default behavior in which suppliers or logistics networks reduce the green index to obtain more economic benefits, resulting in unstable risks in the supply chain network. If the manufacturer is able to “perceive” such defaults, it will impose the necessary penalties on the supplier and the logistics network. When observing that the next trading supplier and logistics network reach the agreed green index, then the manufacturer will choose to continue cooperation, and vice versa. The default node is removed from the supply chain super network, and never cooperates, so that new nodes that meet the green index can be selected to join the supply chain network. Therefore, in order to implement the compliance behavior of the nodes that reach the green contract, manufacturers need to establish certain incentives to improve the green index of suppliers and logistics networks.*

**Hypothesis** **4.** *The manufacturer’s linear demand function is [25]:*

(1) $$D_{s}=D_{l}=\alpha -\beta \left(P_{S}+P_{l}\right)+\lambda \left(e_{s}+e_{l}\right)$$

Among them, *α* represents the parameters of the supply chain super network market size, β represents the demand price elasticity, and λ represents the influence parameter of the supply chain super network green index on the demand, and meets (α,k,λ).

## 3. Construction of the Benefit Allocation Model of a Supply Chain Network

The supply chain network benefits include two parts: one is the income from the interest after the end of each normal transaction, and the other is the value-added profit obtained by improving the green index between the subjects.

**Definition** **1.** *For business networks, suppliers and manufacturers maintain an environmentally friendly contract to maintain the stability and sustainability of the business network, setting $e_{s}\left(0\leq e_{s}\leq 1\right)$ as the green index between supply chain network providers and manufacturers. For logistics networks, manufacturers and third-party logistics companies maintain the stability and sustainability of their supply chain networks by establishing green contracts, setting $e_{l}\left(0\leq e_{l}\leq 1\right)$ as the green index between supply chain network manufacturers and logistics networks.*

**Definition** **2.** *In the process of implementing a green contract, the supplier needs to pay a certain green cost to maintain the green index, so that the supplier’s green cost is $C^{S}\left(e_{s}\right)=\frac{ke_{s}^{2}}{2}$. Similarly, the logistics network needs to pay a certain green cost to maintain the green index, and the logistics network relationship cost is $C^{l}\left(e_{l}\right)=\frac{ke_{l}^{2}}{2}$. Where k>1 represents the supplier network environment cost parameter and meets the following conditions:*

*(1)*  $C^{s}\left(0\right)=C^{l}\left(0\right)=0$ *, indicating that the manufacturer does not incur a relationship cost with the supplier and the logistics network when the green contract is not established.*
*(2)*  $\underset{e_{s}\to 1}{\lim}C^{S}\left(e_{s}\right)=+\infty$ *, which means that when the supplier establishes the maximum green index, the green cost it bears is infinite. For the same reason,* $\underset{e_{l}\to 1}{\lim}C^{l}\left(e_{l}\right)=+\infty$ *indicates that when the logistics network is established to achieve maximum green index, the green cost it undertakes is infinite.*
*(3)*  $\frac{\partial C^{S}\left(e_{s}\right)}{\partial e_{s}}>0,\frac{\partial^{2}C^{S}\left(e_{s}\right)}{\partial e_{s}^{2}}>0$ *, indicating that the supplier’s green index will inevitably lead to an increase in the green cost of the supplier and increase at an increasing rate. Similarly,* $\frac{\partial C^{l}\left(e_{l}\right)}{\partial e_{l}}>0,\frac{\partial^{2}C^{l}\left(e_{l}\right)}{\partial e_{l}^{2}}>0$ *indicates that the logistics network improves the green index, which inevitably leads to an increase in the cost of the logistics network environment.*

**Definition** **3.** *The information flow network will have a positive effect on the stability and benefit distribution of the supply chain network. Let $w_{i}\left(i=1,2,\cdots ,n,0\leq w_{i}\leq 1\right)$ be the information sharing level between the main bodies i of the supply chain network, and it will have a positive effect on the distribution of the interests of the supply chain network. Use $f\left(w_{1},w_{2},\cdots ,w_{n}\right)$ to indicate the positive effect of the information sharing level $w_{i}$ on each subject of the supply chain network.*

### 3.1. Performance Equilibrium Model of Supply Chain Subjects under Green Contracts

The supply chain network observes that after each period of output is realized, if the manufacturer “perceives” that the supplier’s green index is $e_{s}$, and $e_{s}\geq e_{s0}$ ($e_{s0}$ is the lowest level of green index between the manufacturer and the supplier), then the manufacturer will bear a certain proportion of θ supplier green costs. If the manufacturer "perceives" that the green index of the logistics network is $e_{l}$, if $e_{l}\geq e_{l0}$ ($e_{l0}$ is the lowest level of green index between the logistics network and the manufacturer), then the manufacturer will bear a certain proportion of δ (which indicates the logistics network environment costs). Therefore, the manufacturer will coordinate the entire supply chain network to subsidize the performance of suppliers and logistics networks, and then the supplier’s profit function is:

(2) $$\prod_{0}^{u_{1i}}=\left(\omega -c_{1}\right)\left(\alpha -\beta \left(P_{S}+P_{l}\right)+\lambda \left(e_{s}+e_{l}\right)\right)-\frac{\left(1-\theta \right)ke_{s}^{2}}{2}$$

The manufacturer profit function is:

(3) $$\prod_{0}^{u_{2i}}=\left(P_{S}-\omega \right)\left(\alpha -\beta \left(P_{S}+P_{l}\right)+\lambda \left(e_{s}+e_{l}\right)\right)-\frac{\theta ke_{s}^{2}}{2}-\frac{\delta ke_{l}^{2}}{2}$$

Therefore, according to the profit function of the manufacturer and the supplier, the profit function of the business flow network can be found by:

(4) $$\prod_{0}^{S}=\left(P_{S}-c_{1}\right)\left(\alpha -\beta \left(P_{S}+P_{l}\right)+\lambda \left(e_{s}+e_{l}\right)\right)-\frac{ke_{s}^{2}}{2}-\frac{\delta ke_{l}^{2}}{2}$$

The logistics network profit function is:

(5) $$\prod_{0}^{L}=\left(P_{l}-c_{2}\right)\left(\alpha -\beta \left(P_{S}+P_{l}\right)+\lambda \left(e_{s}+e_{l}\right)\right)-\frac{\left(1-\delta \right)ke_{l}^{2}}{2}$$

Therefore, according to the profit function of the business flow network and the logistics network and the utility function of the information flow network, the profit function of the supply chain network can be obtained as:

(6) $$\prod_{0}=\left(P_{S}+P_{l}-c_{1}-c_{2}\right)\left(\alpha -\beta P_{S}+\lambda e_{s}\right)+f\left(w_{1},w_{2},\cdots ,w_{n}\right)-\frac{k\left(e_{s}^{2}+e_{l}^{2}\right)}{2}$$

**Theorem** **1.** *When the manufacturer “perceives” that the green index of each subject is higher than the minimum green index level of the contract, there is a uniquely determined $P_{s}^{*},P_{l}^{*},e_{s}^{*},e_{l}^{*}$ that maximizes the benefits of each subject of the supply chain, and can obtain the best of each subject under the environmental contract. The decision is as follows:*

(7) $$P_{s}^{*}=\frac{\omega -\theta c_{1}}{1-\theta }$$

(8) $$P_{l}^{*}=\frac{\theta \left(\omega -c_{1}\right)\left(1-\delta \right)}{\left(1-\theta \right)\delta }+c_{2}$$

(9) $$e_{s}^{*}=\frac{\lambda \left(\omega -c_{1}\right)}{k\left(1-\theta \right)}$$

(10) $$e_{l}^{*}=\frac{\theta \lambda \left(\omega -c_{1}\right)}{\delta k\left(1-\theta \right)}$$

Proof of the existence of an equilibrium solution. According to the first derivative, the logarithm derivative of the logistics network environment-friendliness $e_{l}$ in the logistics function of the logistics network (5) is obtained and equalized to 0 to obtain $\frac{\partial \Pi_{0}^{L}}{\partial e_{l}}=\lambda \left(P_{l}-c_{2}\right)-\left(1-\delta \right)ke_{l}=0$, thereby obtaining $e_{l}^{\prime }=\frac{\lambda \left(P_{l}-c_{2}\right)}{k\left(1-\delta \right)}$. According to the first derivative, the supplier green index $e_{s}$ and the logistics network green index $e_{l}$ in the manufacturer profit function of Equation (3) are respectively obtained by the partial derivative and equalized to 0 to obtain $\frac{\partial \Pi_{0}^{S}}{\partial e_{s}}=\lambda \left(P_{s}-\omega \right)-\theta ke_{s}=0$, $\;\frac{\partial \Pi_{0}^{S}}{\partial e_{l}}=\lambda \left(P_{s}-\omega \right)-\delta ke_{s}=0$, thereby obtaining $e_{s}^{\prime }=\frac{\lambda \left(P_{s}-\omega \right)}{k\theta },e_{l}^{\prime \prime }=\frac{\lambda \left(P_{s}-\omega \right)}{k\delta }$. Similarly, according to the first derivative, the partial derivative of the supplier green index *e_s_* in Equation (4) is obtained and equalized to 0 to obtain $\frac{\partial \Pi_{0}^{L}}{\partial e_{s}}=\lambda \left(\omega -c_{1}\right)-\left(1-\theta \right)ke_{s}=0$, thereby obtaining $e_{s}^{\prime \prime }=\frac{\lambda \left(\omega -c_{1}\right)}{k\left(1-\theta \right)}$. On this basis, let $e_{s}^{\prime }=e_{s}^{\prime \prime },e_{l}^{\prime }=e_{l}^{\prime \prime }$ find the optimal product pricing $P_{s}^{*}=\frac{\omega -\theta c_{1}}{1-\theta }$ and logistics pricing $P_{l}=\frac{\left(P_{S}-\omega \right)\left(1-\delta \right)}{\delta }+c_{2}$, then substitute $P_{s}^{*}$ into $P_{l}$ to get the optimal logistics pricing $P_{l}^{*}=\frac{\theta \left(\omega -c_{1}\right)\left(1-\delta \right)}{\left(1-\theta \right)\delta }+c_{2}$, and finally substitute the optimal product pricing $P_{s}^{*}$ and the optimal logistics pricing $P_{l}^{*}$ into $e_{s}^{\prime }$ and $e_{l}^{\prime }$. The optimal supplier relationship level $e_{s}^{*}=\frac{\lambda \left(\omega -c_{1}\right)}{k\left(1-\theta \right)}$ and the optimal logistics network relationship level $e_{l}^{*}=\frac{\theta \lambda \left(\omega -c_{1}\right)}{\delta k\left(1-\theta \right)}$ are obtained.

Equilibrium solution uniqueness proof. According to the second derivative pair (5), the logistics pricing *P_l_* in the logistics network profit function and the logistics network green index $e_{l}$ respectively obtain the second-order partial derivative to obtain $\frac{\partial^{2}\Pi_{0}^{L}}{\partial P_{l}^{2}}=-2\beta <0,\;\frac{\partial^{2}\Pi_{0}^{L}}{\partial e_{l}^{2}}=-\left(1-\delta \right)k<0$, that is, the logistics network profit function $\Pi_{0}^{L}$ is a continuous variable of the variables $P_{l}$ and $e_{l}$. This is a concave function, so $P_{l}$ and $e_{l}$ are the only equilibrium solutions for the logistics network. Similarly, according to the second derivative, the product pricing $P_{s}$ in the manufacturer profit function of Equation (3) and the supplier green index $e_{s}$ respectively obtain the second-order partial derivative to obtain $\frac{\partial^{2}\Pi_{0}^{S}}{\partial P_{s}^{2}}=-2\beta <0,\;\frac{\partial^{2}\Pi_{0}^{S}}{\partial e_{s}^{2}}=-\theta k<0$, that is, the business flow network profit function $\Pi_{0}^{S}$ is about the variables $P_{s}$ and $e_{s}$. The continuous dimple function proves that $P_{s}$ and $e_{s}$ are the only equilibrium solutions of the manufacturer’s profit function.

Therefore, under the condition we can see that the manufacturer “perceives” the green index of the supplier and the logistics network meets $e_{s}\geq e_{s0},e_{l}\geq e_{l0}$, the optimal pricing $P_{s}^{*}$ of the supply chain product, the optimal pricing $P_{l}^{*}$ of the logistics, and the optimal green index $e_{s}^{*}$ of the supply chain are obtained, as well as the optimal green index of the logistics network $e_{l}^{*}$. Based on this, Equations (7)–(10) are substituted into the business flow network profit function (4) and the logistics network profit function (5). The optimal profit function of the business flow network and the logistics network can be obtained as follows:

(11) $$\prod_{0}^{S^{*}}=\frac{\left(\omega -c_{1}\right)\left[2k\delta \left(\beta \omega -\beta c_{2}\right)\left(\theta -1\right)+\lambda^{2}\left(\omega -c_{1}\right)\left(\delta -\theta^{2}\right)-2\theta \left(\omega -c_{1}\right)\left(\beta k-\lambda^{2}\right)\right]}{2k\delta {\left(1-\theta \right)}^{2}}$$

(12) $$\prod_{0}^{L^{*}}=\frac{\theta \left(1-\delta \right)\left(\omega -c_{1}\right)\left[k\delta \left(1-\theta \right)\left(\alpha -\beta \left(\omega +c_{2}\right)\right)+\left(\omega -c_{1}\right)\left(\lambda^{2}\left(\delta +0.5\theta \right)-\beta \theta k\right)\right]}{k\delta^{2}{\left(1-\theta \right)}^{2}}$$

Based on the above model analysis, it can be seen that under the condition of fulfilling the relationship contract between the supplier and the logistics network, the overall profit of the supply chain network is as follows:

(13) $$\prod_{0}^{*}=\overset{S^{*}}{\underset{0}{\prod }}+\overset{L^{*}}{\underset{0}{\prod }}+f\left(w_{1},w_{2},\cdots ,w_{n}\right)$$

The following inference can be drawn from the supplier’s optimal green index (9) and the logistics network’s optimal green index (10):

**Inference** **1.** If the supplier and the logistics network maintain a green index which is higher than the manufacturer’s perceived minimum environmental level, then the supplier’s green index and unit product profit $\left(\omega -c_{1}\right)$, the manufacturer’s share of the supplier’s green cost ratio θ, and the green index sensitivity coefficient λ of the demand is positively correlated and negatively correlated with the green cost parameter k. The green index $e_{l}$ of the logistics network is positively correlated with the supplier’s green index $e_{s}$. In addition, the green index $e_{l}$ of the logistics network is also positively correlated with the ratio θδ of the green costs of the supply chain network nodes.

From Inference 1, we know that manufacturers increase the green index by increasing the proportion θ of green costs with suppliers, but the green index is limited by green cost parameters. Excessive green costs will inevitably lead to speculation. This requires manufacturers and suppliers to strengthen coordination to reduce green costs, thereby promoting suppliers to more actively implement green contracts. For the logistics network, the manufacturer and the logistics network need to meet certain conditions to maintain a high green index, that is, the manufacturer assumes that the supplier’s green cost ratio θ is greater than the logistics network environment cost ratio δ. Because the logistics network belongs to an independent third-party logistics enterprise and cannot create the value of the product, it is necessary for the third-party logistics enterprise to find a stable and sustainable supply chain entity. Therefore, when the logistics network observes the supplier and the manufacturer maintaining a high degree of green index will enhance the logistics network’s confidence in the stability of the supply chain, and then share the proportion δ of the service supply chain network at a lower cost.

### 3.2. The Interest Equilibrium Model of a Supply Chain Subject under Speculative Behavior

The supply chain super network observes that after each period of output is realized, the manufacturer “perceives” the supplier’s green index to be $e_{s}$, $e_{s}<e_{s0}$ ($e_{s0}$ is the minimum green index level of the environmental contract between the manufacturer and the supplier), indicating supply. If there is speculation in the business, the manufacturer will make a fine of $\theta \left(e_{s0}-e_{s}\right)$. If the manufacturer “perceives” the green index of the logistics network to be $e_{l}$, $e_{l}<e_{l0}$, indicating that there is speculation in the logistics network, the manufacturer will make a fine of $\delta \left(e_{l0}-e_{l}\right)$. Based on this, the manufacturer will coordinate the entire supply chain network to impose default penalties on suppliers and logistics networks. At this time, the supplier’s profit function is:

(14) $$\prod_{1}^{u_{1i}}=\left(\omega -c_{1}\right)\left(\alpha -\beta \left(P_{S}+P_{l}\right)+\lambda \left(e_{s}+e_{l}\right)\right)-\frac{ke_{s}^{2}}{2}-\theta \left(e_{s0}-e_{s}\right)$$

The manufacturer’s profit function is:

(15) $$\prod_{1}^{u_{2i}}=\left(P_{S}-\omega \right)\left(\alpha -\beta \left(P_{S}+P_{l}\right)+\lambda \left(e_{s}+e_{l}\right)\right)+\theta \left(e_{s0}-e_{s}\right)-\delta \left(e_{l0}-e_{l}\right)$$

Therefore, according to the profit function of the manufacturer and the supplier, the profit function of the business flow network can be found by:

(16) $$\prod_{1}^{S}=\left(P_{S}-c_{1}\right)\left(\alpha -\beta \left(P_{S}+P_{l}\right)+\lambda \left(e_{s}+e_{l}\right)\right)-\frac{ke_{s}^{2}}{2}+\delta \left(e_{l0}-e_{l}\right)$$

The logistics network profit function is:

(17) $$\prod_{1}^{L}=\left(P_{l}-c_{2}\right)\left(\alpha -\beta \left(P_{S}+P_{l}\right)+\lambda \left(e_{s}+e_{l}\right)\right)-\frac{ke_{l}^{2}}{2}-\delta \left(e_{l0}-e_{l}\right)$$

Therefore, according to the profit function of the business flow network and the logistics network and the utility function of the information flow network, the profit function of the supply chain network can be obtained by:

(18) $$\prod_{1}=\left(P_{S}+P_{l}-c_{1}-c_{2}\right)\left(\alpha -\beta \left(P_{S}+P_{l}\right)+\lambda \left(e_{s}+e_{l}\right)\right)+f\left(w_{1},w_{2},\cdots ,w_{n}\right)-\frac{k\left(e_{s}^{2}+e_{l}^{2}\right)}{2}$$

**Theorem** **2.** *When the manufacturer “perceives” that the green index of each subject is lower than the minimum green index level of reaching the contract, there is a uniquely determined $P_{l}^{**},e_{l}^{**},P_{s}^{**},e_{s}^{**}$ that maximizes the returns of each subject of the supply chain, and can obtain the best decision of each subject under speculation, as follows:*

(19) $$P_{l}^{**}=\frac{\theta \left(\omega -c_{1}\right)\left(k+\delta \right)}{\delta \left(k-\theta \right)}+c_{2}$$

(20) $$e_{l}^{**}=\frac{\left(\omega -c_{1}\right)\theta \lambda }{\delta \left(k-\theta \right)}$$

(21) $$P_{s}^{**}=\frac{\omega k-\theta c_{1}}{k-\theta }$$

(22) $$e_{s}^{**}=\frac{\left(\omega -c_{1}\right)\lambda }{k-\theta }$$

Proof of existence of an equilibrium solution. According to the first derivative, the logarithm derivative of the logistics network environment-friendliness $e_{l}$ in the logistics function of the logistics network (17) is obtained and equalized to 0 to obtain $\frac{\partial \Pi_{1}^{L}}{\partial e_{l}}=\lambda \left(P_{l}-c_{2}\right)-\left(k+\delta \right)e_{l}=0$, thereby obtaining $e_{l}^{\prime }=\frac{\lambda \left(P_{l}-c_{2}\right)}{k+\delta }$. According to the first derivative, the supplier green index $e_{s}$ and the logistics network green index $e_{l}$ in the manufacturer profit function of Equation (15) are respectively obtained by the partial derivative and equalized to 0 to obtain $\frac{\partial \Pi_{1}^{S}}{\partial e_{s}}=\lambda \left(P_{s}-\omega \right)-\theta e_{s}=0$, $\frac{\partial \Pi_{1}^{S}}{\partial e_{l}}=\lambda \left(P_{s}-\omega \right)-\delta e_{s}=0$, thereby obtaining $e_{s}^{\prime }=\frac{\lambda \left(P_{s}-\omega \right)}{\theta },e_{l}^{\prime \prime }=\frac{\lambda \left(P_{s}-\omega \right)}{\delta }$. Similarly, according to the first derivative, the partial derivative of the supplier green index $e_{s}$ in Equation (14) is obtained and equalized to 0 to obtain $\frac{\partial \Pi_{1}^{L}}{\partial e_{s}}=\lambda \left(\omega -c_{1}\right)-\left(k-\theta \right)e_{s}=0$, thereby obtaining $e_{s}^{\prime \prime }=\frac{\lambda \left(\omega -c_{1}\right)}{k-\theta }$. On this basis, let $e_{s}^{\prime }=e_{s}^{\prime \prime },e_{l}^{\prime }=e_{l}^{\prime \prime }$ obtain the optimal product pricing $P_{s}^{**}=\frac{\omega k-\theta c_{1}}{k-\theta }$ and logistics pricing $P_{l}=\frac{\left(P_{S}-\omega \right)\left(k+\delta \right)}{\delta }+c_{2}$ under default conditions, then substitute $P_{s}^{**}$ into $P_{l}$ to get the optimal logistics pricing $P_{l}^{**}=\frac{\theta \left(\omega -c_{1}\right)\left(k+\delta \right)}{\delta \left(k-\theta \right)}+c_{2}$, and finally substitute the optimal product pricing $P_{s}^{**}$ and the optimal logistics pricing $P_{l}^{**}$ into $e_{s}^{\prime }$ and $e_{l}^{\prime }$ to get the optimal supplier green index $e_{s}^{**}=\frac{\left(\omega -c_{1}\right)\lambda }{k-\theta }$ and the optimal logistics network green index $e_{l}^{**}=\frac{\left(\omega -c_{1}\right)\theta \lambda }{\delta \left(k-\theta \right)}$.

Equilibrium solution uniqueness proof. According to the second derivative of Equation (17), the logistics pricing $P_{l}$ and the logistics network green index $e_{l}$ respectively obtain the second-order partial derivative to obtain $\frac{\partial^{2}\Pi_{1}^{L}}{\partial P_{l}^{2}}=-2\beta <0,\;\frac{\partial^{2}\Pi_{1}^{L}}{\partial e_{l}^{2}}=-\left(k+\delta \right)k<0$, that is, the logistics network profit function $\Pi_{1}^{L}$ is the continuous variable of the variables $P_{l}$ and $e_{l}$. It is a concave function, so $P_{l}$ and $e_{l}$ are the only equilibrium solutions for the logistics network. Similarly, according to the second derivative, the product pricing $P_{s}$ and the supplier green index $e_{s}$ in the manufacturer profit function of Formula (15) respectively obtain the second-order partial derivative to obtain $\frac{\partial^{2}\Pi_{1}^{S}}{\partial P_{s}^{2}}=-2\beta <0,\;\frac{\partial^{2}\Pi_{1}^{S}}{\partial e_{s}^{2}}=-\theta <0$, that is, the business flow network profit function $\Pi_{1}^{S}$ is about the variables $P_{s}$ and $e_{s}$. The continuous dimple function proves that $P_{s}$ and $e_{s}$ are the only equilibrium solutions of the manufacturer’s profit function.

Therefore, we get the optimal pricing of supply chain products under the condition of default, $P_{s}^{**}$, logistics optimal pricing $P_{l}^{**}$, supply chain optimal environment when the manufacturer “perceives” the green index of suppliers and logistics networks to meet $e_{s}<e_{s0},e_{l}<e_{l0}$. Friendliness is denoted as $e_{s}^{**}$ and the logistics network optimal green index is denoted as $e_{l}^{**}$. Based on this, Equations (19)–(22) are substituted into the business flow network profit function (16) and the logistics network profit function (17) to obtain the optimal profit function of the business flow network and the logistics network in the following when there is default behavior.

(23) $$\prod_{1}^{S^{**}}=\frac{\left(\omega -c_{1}\right)\left[2\delta \left(k\left(\alpha -\beta c_{2}\right)-\theta \lambda \right)\left(k-\theta \right)-2\beta k\left(\omega k\left(\delta +\theta \right)+\theta \delta \left(\omega -2c_{1}\right)-k\theta c_{1}\right)+\lambda^{2}k\left(\omega -c_{1}\right)\left(\delta +2\theta \right)\right]}{2\delta {\left(k-\theta \right)}^{2}}+\delta e_{l0}$$

(24) $$\prod_{1}^{L^{*}}=\frac{\left(\omega -c_{1}\right)\left[\begin{array}{c} \left(k+\delta \right)\left(2\theta \delta \left(k-\theta \right)\left(\alpha -\beta c_{2}\right)-2\beta \theta^{2}\left(\delta \omega -2c_{1}\delta -c_{1}k\right)+2\theta \lambda^{2}\left(\omega -c_{1}\right)\left(\delta +\theta \right)-2\beta \theta \omega k\left(1+\theta \right)\right)\\ -k\theta^{2}\lambda^{2}\left(\omega -c_{1}\right)+\theta \lambda \delta^{2}\left(k-\theta \right) \end{array}\right]}{2\delta^{2}{\left(k-\theta \right)}^{2}}-\delta e_{l0}$$

Based on the above model analysis, it can be seen that under the condition of default behavior of suppliers and logistics networks, the overall profit of the supply chain network is as follows:

(25) $$\prod_{1}^{**}=\overset{S^{**}}{\underset{1}{\prod }}+\overset{L^{**}}{\underset{1}{\prod }}+f\left(w_{1},w_{2},\cdots ,w_{n}\right)$$

If there is speculation in the supplier and logistics network, the following inference can be drawn from the logistics network green index (20) and the supplier’s optimal green index (22):

**Inference** **2.** If suppliers and logistics networks maintain the green index below the minimum green index which manufacturers perceive, the suppliers’ green index and unit product profit $\left(\omega -c_{1}\right)$, manufacturer’s penalties θ for suppliers, and green index sensitivity λ to the demand are positive correlation and negative correlation with the green cost parameter k. The green index $e_{l}$ of the logistics network is positively correlated with the supplier’s green index $e_{s}$, and at the same time it is also positively correlated with the manufacturer’s ratio θδ of the penalty power of the supply chain network nodes.

From Inference 2, we know that, on the one hand, manufacturers can increase the green index of suppliers by increasing the penalty θ for supplier defaults, but the excessive punishment will hinder the supplier’s enthusiasm for fulfilling the contract and they may even exit the supply chain network. The stability of the manufacturer and the entire supply chain network is risky, so reasonable penalties are beneficial to improve supplier compliance. On the other hand, the green index is restricted by the green cost parameter *k*. The existence of a green cost is a prerequisite for suppliers to speculate. The only way for suppliers to reduce green costs is to fulfill the green contract with the manufacturers. For the logistics network, its green index will be affected by the green index of the supplier. As the third-party logistics companies observe that the supplier has defaulted, the green index level of the supply chain business network will be reduced. The improvement of the green index of the logistics network will be negative, so the logistics network will also have a lower green index.

**Inference** **3.** *If the manufacturer “perceives” that the supplier and logistics network green index meets $e_{s}\geq e_{s0},e_{l}\geq e_{l0}$, its supply chain network will earn a profit $\Pi_{0}^{**}$. If the manufacturer “perceives” that the supplier and logistics network green index meet $e_{s}<e_{s0},e_{l}<e_{l0}$, and its supply chain network gets a profit $\Pi_{1}^{**}$, then $\Pi_{0}^{**}>\Pi_{1}^{**}$.*

**Proof.**  For the sake of calculation, we convert Equation (11) into $\frac{\left(\omega -c_{1}\right)*A}{2K\delta {\left(1-\theta \right)}^{2}}$, convert Equation (12) into $\frac{\left(\omega -c_{1}\right)*B}{K\delta^{2}{\left(1-\theta \right)}^{2}}$, convert Equation (23) into $\frac{\left(\omega -c_{1}\right)*C}{2\delta {\left(K-\theta \right)}^{2}}$, and convert Equation (24) into $\frac{\left(\omega -c_{1}\right)*D}{\delta^{2}{\left(K-\theta \right)}^{2}}$. Because $k\geq 1,\frac{\omega -c_{1}}{2K\delta {\left(1-\theta \right)}^{2}}\geq \frac{\omega -c_{1}}{2\delta {\left(K-\theta \right)}^{2}}$, and $\frac{\omega -c_{1}}{K\delta^{2}{\left(1-\theta \right)}^{2}}\geq \frac{\omega -c_{1}}{\delta^{2}{\left(K-\theta \right)}^{2}}$ can be obtained. Therefore, we only need to ask A+B−(C+D)>0 to establish this, so that we can get:

$$\begin{array}{cc} \Pi_{0}^{**}-\Pi_{1}^{**} & =k\beta \delta \left(1-\theta \right)\left(c_{2}+2\omega \right)+\left(\omega -c_{1}\right)\left(\lambda^{2}\left(\left(3+k-\theta \right)\theta +2\delta +k\left(2\theta +\delta +\beta \theta \right)\right)+2k^{2}\theta \beta \right)\\ & +\alpha \theta \delta \left(2-k\right)+k\delta \left(\alpha -\omega \beta \right)\left(1+2k\right)+\lambda \delta^{2}\left(k-\theta \delta \right)+2\delta \omega \beta \theta +2\delta \beta c_{2}k\left(k-2\theta \right)\\ & +2k\delta \left(\beta c_{2}+\alpha \right)+\beta \theta \delta \left(\omega k-2c_{1}\right)+2\delta \theta \lambda \left(k-\theta \right)+2k\omega \beta \theta \left(1-\delta \right)+2\beta \theta kc_{1}\left(1-2\delta \right)\\ & >0 \end{array}$$

s.t k≥1,0≤θ≤1,0≤δ≤1,α−βω>0, this can be proved by $\Pi_{0}^{**}>\Pi_{1}^{**}$.  □

According to Inference 3, suppliers and logistics networks maintain a high level of green index. The overall benefits of the supply chain network are greater than the low-level green supply chain network benefits under both speculative conditions. Therefore, maintaining a high green index of the supply chain entity is beneficial to the benefits of the supply chain network, not only to satisfy the profitability of the supply chain, but also to achieve sustainability of the supply chain.

**Inference** **4.** *Maintaining a green index for suppliers and logistics networks requires green costs, while green costs are constrained by the green cost parameter k. On the one hand, regardless of whether the green index of suppliers and logistics networks reaches the minimum green index level that manufacturers “perceive”, with the increase of green cost parameter k, their green index, business network profit and logistics network profit are marginal. On the other hand, under the condition of performance, the product price and the unit price of the logistics are not affected by the green cost parameter k. Under the condition of default, the product price and the unit price of the logistics will decrease with an increase of the green cost parameter k.*

**Proof.**  Under the condition of fulfilling the relationship contract, the supply chain entity separately obtains the partial derivative of the relationship cost parameter *k* from the decision variable $P_{s}^{*},P_{l}^{*},e_{s}^{*},e_{l}^{*},\Pi_{S}^{*},\Pi_{L}^{*}$ to obtain $\frac{\partial P_{s}^{*}}{\partial k}=0,\frac{\partial P_{l}^{*}}{\partial k}=0,\frac{\partial e_{s}^{*}}{\partial k}=-\frac{\lambda \left(\omega -c_{1}\right)}{\left(1-\theta \right)k^{2}}<0,\frac{\partial e_{l}^{*}}{\partial k}=-\frac{\theta \lambda \left(\omega -c_{1}\right)}{\left(1-\theta \right)k^{2}\delta }<0$, $\frac{\partial \Pi_{S}^{*}}{\partial k}=-\frac{\theta \lambda^{2}{\left(\omega -c_{1}\right)}^{2}\left(\delta +2\theta -\theta^{2}\right)}{{\left(1-\theta \right)}^{2}k^{2}\delta }<0,\frac{\partial \Pi_{L}^{*}}{\partial k}=-\frac{\theta \lambda^{2}{\left(\omega -c_{1}\right)}^{2}\left(\delta +0.5\theta \right)\left(1-\delta \right)}{{\left(1-\theta \right)}^{2}k^{2}\delta^{2}}<0$. Under the condition of default, the supply chain entity separately obtains the partial derivative of the relationship cost parameter k from the decision variable $P_{s}^{**},P_{l}^{**},e_{s}^{**},e_{l}^{**},\Pi_{S}^{**},\Pi_{L}^{**}$ to obtain $\frac{\partial P_{s}^{**}}{\partial k}=-\frac{\theta \left(\theta +\delta \right)\left(\omega -c_{1}\right)}{{\left(k-\theta \right)}^{2}\delta }<0,\frac{\partial P_{l}^{**}}{\partial k}=-\frac{\theta \left(\omega -c_{1}\right)}{{\left(k-\theta \right)}^{2}}<0,\frac{\partial e_{s}^{**}}{\partial k}=-\frac{\lambda \left(\omega -c_{1}\right)}{{\left(k-\theta \right)}^{2}}<0,\frac{\partial e_{l}^{**}}{\partial k}=-\frac{\theta \lambda \left(\omega -c_{1}\right)}{{\left(k-\theta \right)}^{2}\delta }<0$ and $\frac{\partial \Pi_{S}^{**}}{\partial k}=-\frac{\left(\omega -c_{1}\right)\left(\lambda^{2}\left(\omega -c_{1}\right)\left(\delta +2\theta \right)\left(\theta +k\right)\right)+2\delta \theta^{2}\left(\lambda +\beta c_{2}\right)}{2{\left(k-\theta \right)}^{3}\delta }<0,\frac{\partial \Pi_{L}^{**}}{\partial k}=-\frac{\lambda^{2}\theta {\left(\omega -c_{1}\right)}^{2}\left(\theta^{2}+2\delta^{2}+6\theta \delta +2k\theta \right)}{{\left(k-\theta \right)}^{3}\delta^{2}}<0$.  □

As can be seen from Inference 4, under the condition that the supply chain entity fulfills the green contract, the manufacturer can reduce the green cost parameter *k* with the supply chain and the logistics network through technology transfer and capital support, thereby improving the overall income level of the supply chain. The condition of speculative behavior in the supply chain, due to the increase of the green cost parameter *k*, can only lead to environmentally unfriendly behaviors of suppliers and logistics networks. On the one hand, suppliers increase the supply of environmentally unfriendly products, and the greenness of the overall product is reduced, which leads to a decrease in the selling price of the product. On the other hand, the logistics network will inevitably reduce the unit price of the logistics due to the cost reduction, but this has a loss for the profit and sustainability of the overall supply chain network.

In summary, we can draw two conclusions. First, the green development of the supply chain network can increase the income of the main body of the supply chain and the supply chain network, which not only satisfies the principle of maximizing the interests of the supply chain, but also realizes the sustainability of the supply chain to adapt to the development of the times. Second, the various entities within the supply chain network should work together to reduce the impact of green cost parameters on the green index and supply chain benefits.

## 4. Numerical Simulation Analysis

Based on the parameter settings of the literature [26], the numerical results of the study are used to verify the results and inferences in the text, assuming the parameters $\omega =50,c_{1}=40,c_{2}=10,k=140,\alpha =100,\beta =0.5,\lambda =1.3$, and the positive utility $f\left(w_{1},w_{2},\cdots ,w_{n}\right)=1$ in the information flow network due to the information sharing of each subject. At the same time, the minimum green index set by the manufacturer is $e_{s0}=e_{l0}=0.3$, which allows the manufacturer to bear or punish at the proportion θ=0:0.1:1 to the supplier’s green costs, so that the manufacturer assumes or punishes at the proportion δ=0:0.1:1 to the green cost of the logistics network, and then substitutes the relevant parameters into the article model. Using the maple 18.0 software to calculate if and only when θ=0.7,δ=0.6, the overall interests of the supply chain network entities and the supply chain network reach the maximum. At this time, the relevant decision variables of the supply chain network entities in fulfilling the environmental contract and violating the environmental contract are obtained. The interests of the various entities are shown in Table 1 and Table 2 below:

From Table 1 and Table 2 we see, on the one hand, the higher the green index, the greater the gain; on the other hand, the gains from the fulfillment of the supply chain network are much greater than the supply chain network revenue under default conditions, which intuitively indicates that the supply chain network is from top to bottom. The improvement of the supply chain environment is beneficial to the development of the supply chain network, which also verifies the accuracy of Inference 3. At the same time, it also conveys an important message to the main body of the supply chain, that is, it needs to focus on the long-term, and not only be concerned with current interests and its own gains and losses, and continuously improving the green index of products and production processes is beneficial to its own development.

### 4.1. Manufacturer’s Behavior θ,δImpact on Green Index

**Figure 2:**
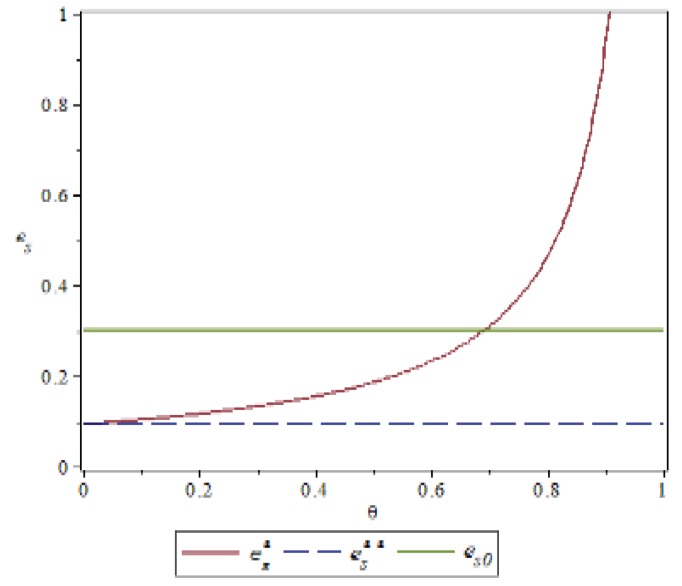
Effect of manufacturer’s behavior *θ* on the supplier green index.

**Figure 3:**
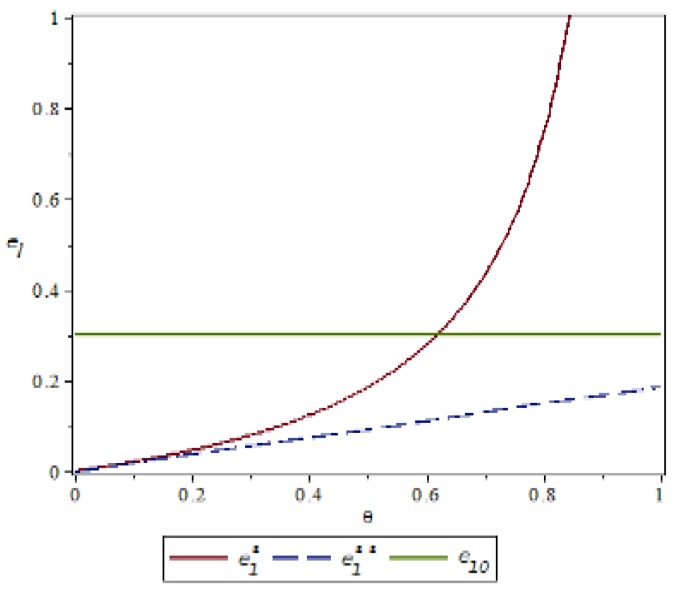
Effect of manufacturer’s behavior *θ* on the green index of logistics networks.

**Figure 4:**
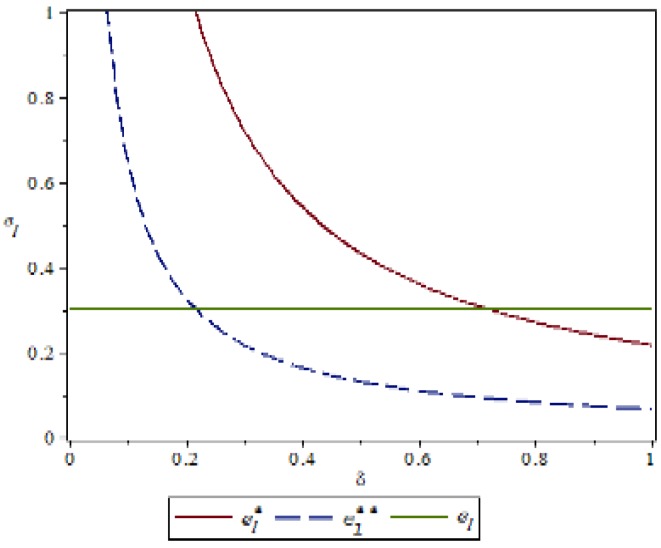
The effect of manufacturer’s behavior *δ* on the green index of logistics networks.

**Figure 5:**
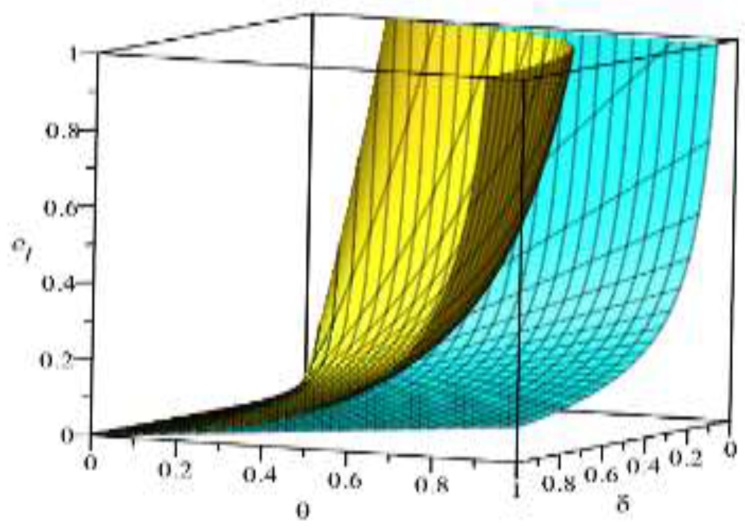
The effect of manufacturer’s behavior θ,δ on the green index of logistics networks.

Based on the above parameter settings, the trend graph of the influence of manufacturer behavior θ,δ on supplier and logistics network environmental performance under contract or speculative conditions is as follows:

Figure 2 and Figure 3 show that manufacturers can increase the green cost sharing ratio θ under compliance conditions, which can improve the green index of suppliers and logistics networks. Under the condition of default, the manufacturer can increase the penalty for the manufacturer θ, which can also improve the green index of the supplier and the logistics network, but the green index of the logistics network is more obvious. This is because the logistics network observes the manufacturer’s increase in supplier penalties, with a huge speculative cost, which may reflect the risk of the manufacturer removing the supply chain network. It can be concluded that under the conditions of compliance, in order to improve the overall green index of the supply chain network, manufacturers should expand the proportion of green cost sharing without compromising their own interests. On the contrary, under speculative conditions, manufacturers can pass reasonable punishment to improve the green index of the supply chain network. Figure 4 shows that under the contract condition, as the manufacturer increases the logistics network environment cost sharing ratio δ, the green index of the logistics network is reduced. This is because the logistics network itself does not create value, and the green behavior it generates only reflects the transportation of green products, and there is an upper limit to the improvement of the green index. Compared to the increase in the proportion of cost of the logistics network environment, it is difficult to improve the green index of the logistics network, so the green index of the logistics network will show a relative downward trend. In Figure 5, the upper layer represents the trend of compliance behavior, and the lower layer represents the trend graph of speculative behavior. From the figure, we can see that under the contract conditions, the manufacturer expands the green cost sharing ratio θ,δ of the supplier and the logistics network, which can improve the logistics network green index, and when there is speculation in the logistics network, the manufacturer can increase the green index of the logistics network by increasing the penalty θ for the supplier or reducing the penalty δ for the logistics network. This also verifies the accuracy of Inference 1 and Inference 2.

### 4.2. The Influence of Manufacturer’s Behavior θ,δ on the Revenue of the Supply Chain Sub-network

**Figure 6:**
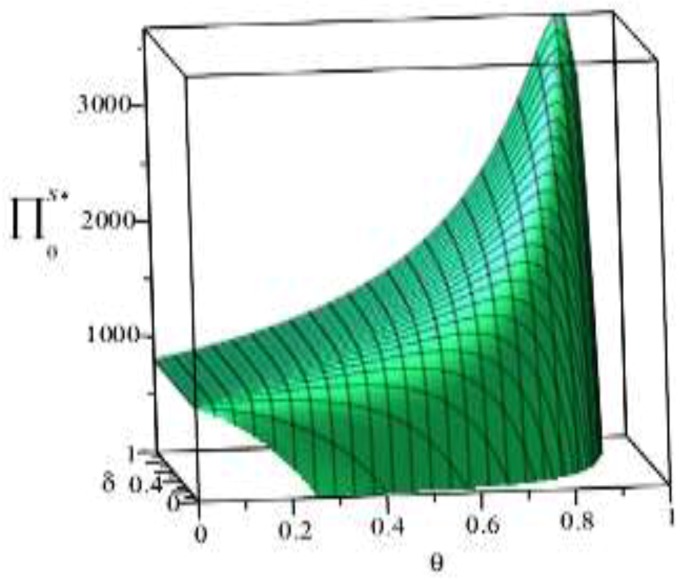
Effect of manufacturer’s behavior θ,δ on business flow network under performance conditions.

**Figure 7:**
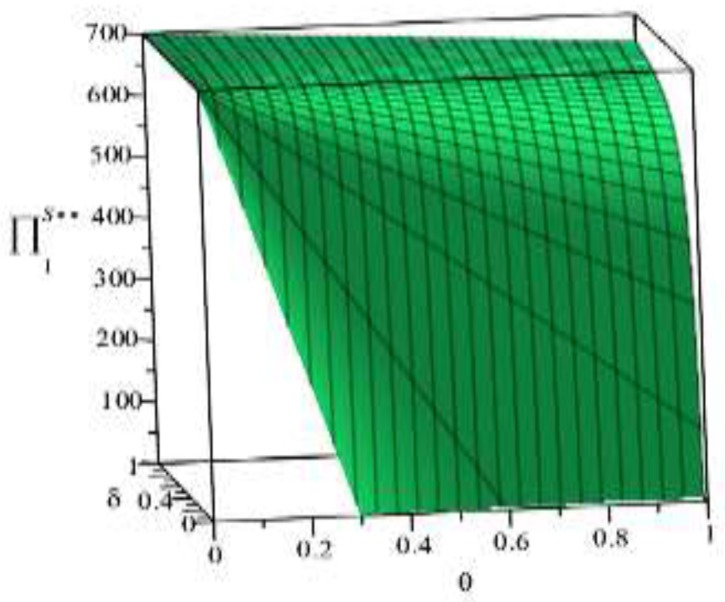
Effect of manufacturer’s behavior θ, δ on business flow network under speculative conditions.

**Figure 8:**
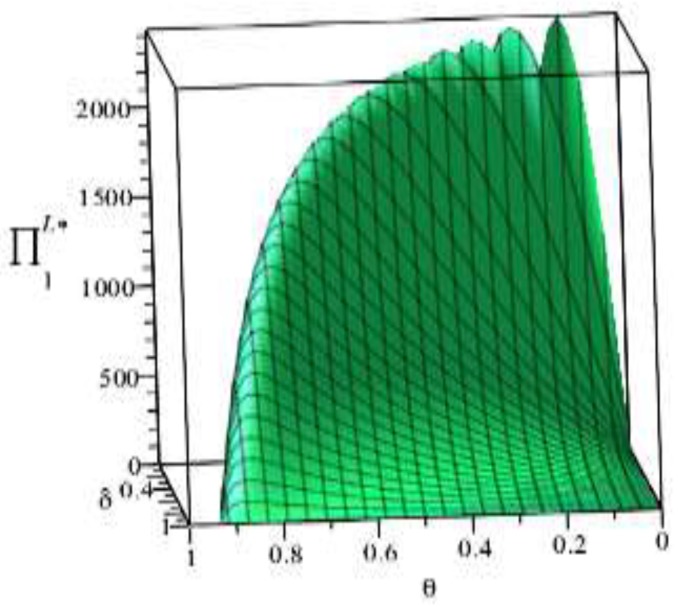
Effect of manufacturer’s behavior θ, δ on logistics network under performance.

**Figure 9:**
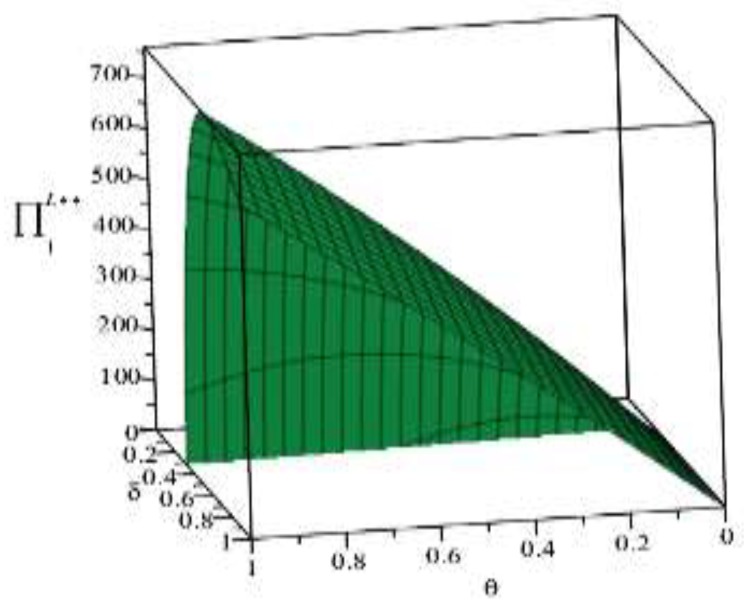
Effect of manufacturer’s behavior θ, δ on logistics network under speculative conditions.

Based on the above parameter settings, the trend graph of the influence of manufacturer’s behavior θ, δ on the business flow network and logistics network revenue under performance or speculative conditions is as follows:

Combining the trend graph of the above-mentioned manufacturer’s behavior on the network profit, combined with the article related theorem and the decision model setting, the following four conclusions can be drawn:

(1) As can be seen from Figure 6, under the condition of fulfilling the environmental contract, if the manufacturer’s green cost commitment ratio θ of the supplier is kept unchanged, the manufacturer’s green cost commitment ratio δ. to the logistics network is simply increased. The profit level of the streaming network shows a trend of rising sharply and then slowly decreasing. If the manufacturer’s green cost commitment ratio δ of the logistics network is kept unchanged, simply increasing the manufacturer green cost commitment ratio θ to the supplier, the profit level of the business flow network firstly rises slowly and then drops sharply. This phenomenon indicates that the manufacturer’s green cost commitment ratio is too high or too low and will have an adverse impact on the profit of the business network, and the intersection of these two phenomena is the optimal interval of the manufacturer’s green cost commitment ratio θ, δ.

(2) Figure 7 shows that, on the one hand, the overall profit level of the commercial network under speculative conditions is lower than the profit level of the commercial network at the time of performance, which is consistent with the inference of the article. On the other hand it can be seen that the logistics network is appropriate. The punishment can increase the profit level of the business network in the short term, but there is an upper limit on the punishment of the logistics network. Once this limit is exceeded, the profit level of the business network will not improve, but the third-party logistics enterprise will be weak. The risk of exiting the supply chain network should be taken. Therefore, reasonable penalties are beneficial to improve the green index and profitability of the supply chain.

(3) Figure 8 shows that, under the condition of fulfilling the environmental contract, if the manufacturer’s environment cost-sharing ratio δ of the logistics network is kept unchanged, then increasing the manufacturer’s green cost-share ratio θ of the supplier can improve the logistics network profit level. On the contrary, if the manufacturer’s green cost-share ratio θ of the supplier is kept constant, then increasing the manufacturer’s share ratio δ of the green cost of the logistics network makes the profit level of the logistics network decline. Because of the logistics unit price and because the green cost-sharing ratio δ is inversely proportional, when the manufacturer increases the green cost-sharing ratio δ, the logistics unit price is closer to the logistics unit cost. When the green cost-sharing ratio δ=1, the logistics unit price is equal to the logistics unit cost, that is $p_{l}=c_{2}$, and then the logistics network profit is 0. Therefore, maintaining a reasonable cost-sharing ratio is beneficial to the profitability of the logistics network.

(4) Figure 9 shows that, on the one hand, the overall profit level of the logistics network under speculative conditions is lower than the profit level of the logistics network at the time of performance, and this trend is in line with the inference of Article 3. On the other hand, under the speculative conditions, the maximum profit point of the logistics network appears at (θ,δ)=(1.0,0.6). Based on this point, reducing or increasing the penalties of the manufacturer for the logistics network will reduce the profit level of the logistics network. At the same time, it can be seen that the manufacturer reducing the penalty θ for suppliers will directly lead to the continuous profit level of the logistics network, which reflects the improvement of the green index of the logistics network and the fulfillment of the environmental contract. Therefore, under speculative conditions, manufacturers can improve the profitability of the logistics network and green index by increasing the penalties imposed on manufacturers.

### 4.3. Influence of Manufacturer’s Behavior θ,δ on Supply Chain Network Revenue

**Figure 10:**
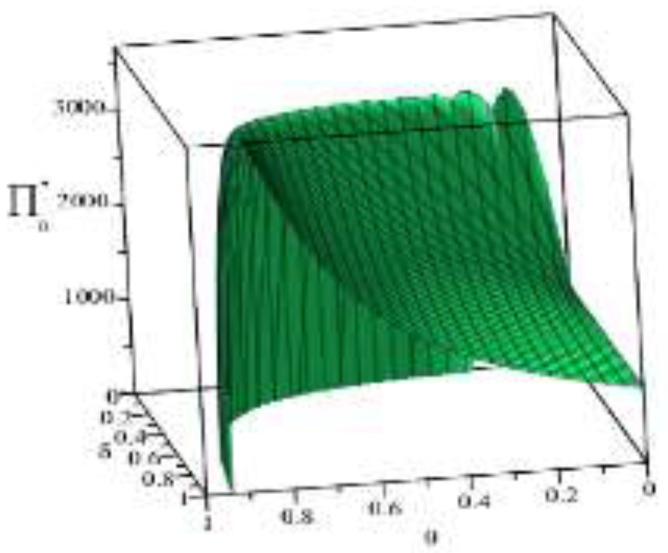
Effect of manufacturer behavior A on a supply chain network under performance conditions.

Based on the above parameter settings, the trend graph of the influence of manufacturer behavior θ,δ on the overall revenue of the supply chain network under performance or speculative conditions is as follows:

According to Figure 10, we can draw the following conclusions under the conditions of fulfilling the environmental contract. On the whole, the maximum value of the supply chain network appears at θ=δ, and at the same time the manufacturer’s green cost- share ratio θ,δ for suppliers and logistics networks is increased, which can effectively improve the overall income level of the supply chain network. From a partial point of view, if the manufacturer’s green cost-commitment ratio θ of the supplier is kept unchanged, as the manufacturer’s green cost-commitment ratio δ of the logistics network increases, it can be seen that the profit level of the business network first rises sharply. The trend of slow decline is because the green cost-sharing ratio δ will continue to increase after a certain interval, which will lead to the unit logistics price getting closer to the unit logistics cost, thus reducing the logistics network profit. If the manufacturer’s green cost-commitment ratio δ of the logistics network is kept unchanged, when θ≤δ, the supply chain network revenue increases with the increase of θ. When θ>δ, the supply chain network revenue decreases with the increase of θ.

**Figure 11:**
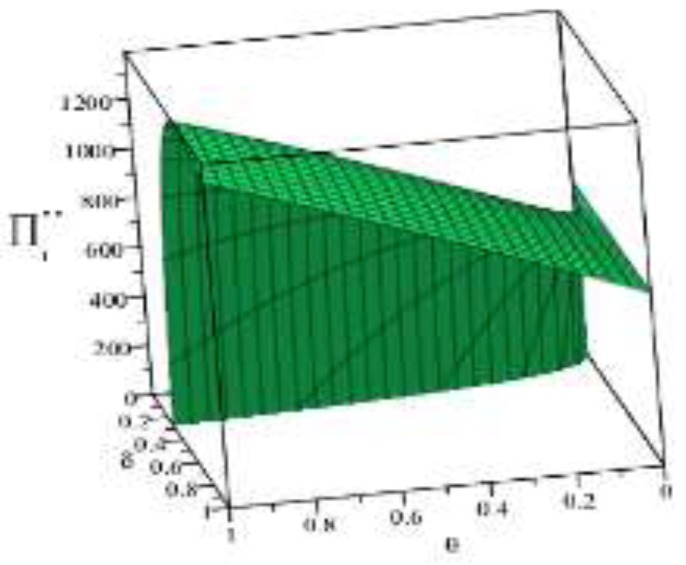
Effect of manufacturer behavior A on supply chain network under speculative conditions.

According to Figure 11, under the conditions of speculation, we can draw the following conclusions. Manufacturers increase speculative penalties for suppliers and improve the overall income level of the supply chain network. At the same time, in order to protect the revenue of the supply chain network, the proper punishment of the logistics network can also improve the overall income of the logistics network. However, excessive punishment will have negative effects on the supply chain network revenue, which is not conducive to the cooperation of network entities in the next transaction. Therefore, manufacturers’ appropriate penalties for speculation are beneficial for maintaining supply chain network revenue and the green index.

### 4.4. Impact of Green Cost Parameter k on Supply Chain Network Revenue

**Figure 12:**
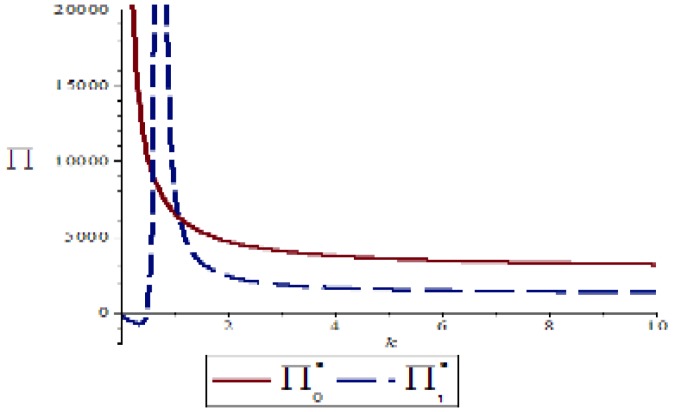
Effect of green cost parameter *k* on the revenue of supply chain networks under different conditions.

Based on the above parameter settings, let θ=0.7,δ=0.6 discuss the impact of green cost parameter *k* on the overall revenue of the supply chain network under performance or speculation conditions as follows:

Figure 12 shows that a supply chain entity exhibits an inverse proportional relationship between the supply chain profit function and the green cost parameter k in the performance of environmental contract or speculative behavior, which also verifies the accuracy of Inference 4. Therefore, in order to improve the profit level of the supply chain network, another effective way is to actively reduce the green cost parameter k, which not only can improve the green index of the supply chain entity, but also avoid the speculative behavior of the supply chain entity due to the existence of green costs. To further promote the stability of the supply chain network, it can also be concluded from the trend graph that, as the article assumes the green cost parameter k≥1, regardless of the change of the cost parameter, the supply chain network profit generated under the performance condition is always greater than the profit of the supply chain network under speculative conditions, which reflects the important impact of fulfilling the contract on the promotion of the supply chain entity.

## 5. Conclusions

This paper tries to link the different network entities of a supply chain and green index to represent the sustainability of a supply chain network. Under the principle of maximizing the benefits of the supply chain network, we hope to see its green development, which can not only realize the economic benefits of the supply chain network, but also meet the social benefits, so as to achieve a win–win effect. Therefore, this paper establishes a supply chain network model that integrates business flow networks, logistics networks and information flow networks. In order to study the sustainability of the supply chain network, the green index is introduced to represent the sustainable development of the supply chain network for the first time, and the benefits of the supply chain network are compared by the performance of a green contract and the existence of speculative behavior. It is concluded that the main body of the supply chain can obtain more benefits by fulfilling a green contract, which is of great significance for the main body of the supply chain to reach a green development consensus. On the other hand, this paper studies the impact of a green index on the balance of interests of the supply chain network, and considers the profitability and sustainability of the supply chain network under the conditions of fulfilling green contract or speculative behavior. Finally, the accuracy and scientific rigor of the conclusions are verified by numerical simulation analysis. Meanwhile considering the manufacturer-led situation, this paper explored the relationship between manufacturer behavior θ,δ and the green cost parameter k on the supply chain network decision variables and network revenue models. In summary, we draw the following conclusions and recommendations:

(1) Under the conditions of compliance, the dominant manufacturer can improve the green index of suppliers and logistics networks by increasing the proportion of green cost to suppliers θ, and the green index of the logistics network is related to the manufacturer. The logistics network green cost-sharing ratio δ is inversely proportional, so the green cost-sharing ratio δ should be selected according to the actual situation. Under speculative conditions, the dominant manufacturer can restrain the supplier and the logistics network from fulfilling the environmental contract by increasing the penalty ratio θ to the supplier, thereby improving the green index. For the logistics network, a greater penalty ratio δ instead will lead to a decline in the green index of the logistics network, so manufacturers can motivate the logistics network to fulfill the environmental contract by punishing the supplier and appropriately punishing the logistics network.

(2) Under the conditions of compliance, increasing the cost-sharing ratio of the manufacturer θ,δ can increase the profit level of the supply chain network. On the contrary, manufacturers can increase the speculative penalty ratio θ of suppliers and reduce the logistics network speculative penalty ratio δ to increase the profit level of the supply chain network under speculative conditions.

(3) The existence of green cost is the main factor of speculative behavior in the supply chain, while the green cost parameter k is related to the green cost. The article shows that with the increase of green cost parameter k, the supply chain network revenue shows a downward trend. Therefore, reducing the green cost parameter k is the main direction for the supply chain entities to achieve sustainable development of the supply chain network. Manufacturers can reduce the green cost parameter *k* of the supply chain partners through technology sharing and financial support. The overall improvement of the green index of the chain network improves the profit level of the supply chain network.

(4) For business flow networks, logistics networks and supply chain networks, fulfilling environmental contracts is positive for improving network interests, and there are more additional benefits obtained by supply chain owners than speculative behaviors. If there is an act of reducing interest, the supply chain entity should actively participate in the green development of the supply chain network according to the actual situation and its own factors, and realize the profitability and sustainability of the supply chain network.

This article compares and analyzes the coordination of interests in the supply chain entity’s performance or violation of the green contract, and concludes that fulfilling the green contract is beneficial to the supply chain network. It studies the influence and advantage of the manufacturer’s behavior θ,δ on the sustainable development of the supply chain, and from the theory, the feasibility of this is demonstrated. This article enriches the research of supply chain networks in the field of environmental sciences, and provides reference to the relevant decision-making in the sustainable development of a supply chain. However, this paper only proves the validity and scientific rigor of the method and model in theory. Although the experimental simulation is given, the model has not been applied in actual scenarios and lacks the support of actual background data. Therefore, in the next step of the research, it is necessary to combine the actual background for analysis and research. At the same time, uncertain risk should be considered as an impact supply in subsequent research. In order to meet the requirement of a green supply chain to adapt to a complex environment, the influence decision variables of maximum benefit and sustainable development should be introduced into the model.

## Figures and Tables

**Table 1:** Supply chain network benefit equilibrium variables under compliance conditions.

$q_{s}^{*}$	$e_{s}^{*}$	$q_{l}^{*}$	$e_{l}^{*}$	$\Pi_{0}^{S*}$	$\Pi_{0}^{L*}$	$\Pi_{0}^{*}$
73.3300	0.3095	25.56	0.3611	2035.3960	796.3302	2832.7260

Note: S-Business flow network; L-Logistics network; *: means optimal solution under compliance conditions.

**Table 2:** Supply chain network benefit equilibrium variables under speculative conditions.

$q_{s}^{**}$	$e_{s}^{**}$	$q_{l}^{**}$	$e_{l}^{**}$	$\Pi_{1}^{S**}$	$\Pi_{1}^{L**}$	$\Pi_{1}^{**}$
50.0502	0.0930	21.77	0.1088	646.2383	559.5334	1206.7720

Note: S-Business flow network; L-Logistics network; **: means optimal solution under speculative conditions.
